# Characteristics of ischemic stroke and intracranial hemorrhage in patients with nephrotic syndrome

**DOI:** 10.1186/s12882-021-02415-w

**Published:** 2021-06-05

**Authors:** Wen-Yi Huang, Chun-Wei Chang, Chiung-Mei Chen, Kuan-Hsing Chen, Chien-Hung Chang, Hsiu-Chuan Wu, Kuo-Hsuan Chang

**Affiliations:** 1 Department of Neurology, Chang Gung University College of Medicine, Chang Gung Memorial Hospital, Keelung Branch, No.222, Mai-Jin Road, 204 Keelung, Taiwan; 2 Department of Neurology, Chang Gung University College of Medicine, Chang Gung Memorial Hospital, Linkou Branch, 5, Fu Hsing Street, 333 Taoyuan, Taiwan; 3 Kidney Research Center, Chang Gung Memorial Hospital, Linkou Branch, Chang Gung University, School of Medicine, 5, Fu Hsing Street, Taoyuan, 333 Taiwan

**Keywords:** Nephrotic syndrome, Stroke, Intracranial hemorrhage, Ischemic stroke, Intracerebral hemorrhage, Subarachnoid hemorrhage

## Abstract

**Background:**

The incidence of cerebral stroke, including ischemic infarction and intracranial hemorrhage (ICH), increases in patients with nephrotic syndrome (NS). However, the clinical characteristics of patients with NS and stroke remain elusive. We aimed to investigate the clinical presentation and prognosis among patients with NS and ischemic stroke (IS) or ICH.

**Methods:**

We conducted a population-based retrospective cohort study of patients with NS and acute stroke using the Chang Gung Research Database of Taiwan from January 1, 2001, to December 31, 2017. The participants were recruited from the 7 branches of Chang Gung Memorial Hospital.

**Results:**

A total of 233 patients with IS and 57 patients with ICH were enrolled. The median age was 60 (52–70) years. The prevalence rates of hyperlipidemia, hyperuricemia, and smoking were higher in IS than in ICH. IS demonstrated lower white blood cell count (7.80 vs. 8.92 × 10^9^/L) and high-sensitivity C-reactive protein level (33.42 vs. 144.10 nmol/L) and higher cholesterol (5.74 vs. 4.84 mmol/L), triglyceride (1.60 vs. 1.28 mmol/L), and albumin (24 vs. 18 g/L) levels compared with ICH. The dependent functional status and 30-day mortality were higher in ICH than in IS. The risk factors for 30-day mortality for patients with NS and stroke were coronary artery disease (CAD), ICH, and total anterior circulation syndrome. The multivariate Cox regression analysis revealed that CAD was positively associated with 30-day mortality in patients with IS (hazard ratio 24.58, 95 % CI 1.48 to 408.90). In patients with ICH, CAD and subarachnoid hemorrhage were positively associated with 30-day mortality (hazard ratio 5.49, 95 % CI 1.54 to 19.56; hazard ratio 6.32, 95 % CI 1.57 to 25.53, respectively).

**Conclusions:**

ICH demonstrated a higher risk of dependence and 30-day mortality compared with IS in patients with NS. Intensive monitoring and treatment should be applied particularly in patients with NS and ICH.

**Supplementary Information:**

The online version contains supplementary material available at 10.1186/s12882-021-02415-w.

## Background

Nephrotic syndrome (NS) is characterized by the presence of proteinuria, peripheral edema, hypoalbuminemia, and increased risk of both venous and arterial thromboses. An increased risk of cardiovascular diseases has been reported in patients with NS [1]. The hypercoagulable state of patient with NS results from an imbalance between procoagulant/prothrombotic and anticoagulant/antithrombotic factors, which promotes in situ thrombosis in the deep veins or arteries [1, 2]. Although arterial thrombosis occurs mostly in children, the occurrence of strokes, especially ischemic stroke (IS), is not uncommon in adult patients with NS [3, 4]. Several case reports [3–6] and retrospective cohort studies [7, 8] suggest that patients with NS have an increased risk of IS and venous sinus thrombosis.

On the other hand, intracranial hemorrhage (ICH) is also seen in patients with NS [9–11]. This cerebral hemorrhage, which could be associated with intravascular deposition of immune complexes or systemic amyloidosis [12–14], can occur in patients with NS without stroke risk factors [11, 15, 16]. Biochemical abnormalities, such as proteinuria, D-dimer levels, hyperlipidemia, and renal function impairment, are associated with the occurrence of ICH in patients with NS [11]. However, the small numbers of patients in these studies limit further understanding of the clinical outcome and potential pathogenesis in this subpopulation of patients.

Although lines of evidence suggest that the incidence rates of IS and ICH both increase in patients with NS, research examining the functional outcome differences in those with IS versus ICH is limited. It would be important to understand the clinical presentations and outcomes of these patients with both types of stroke by a large cohort study. Using real-world data from the Chang Gung (CG) Research Database, we conducted a retrospective cohort study to investigate the clinical presentations of NS patients with stroke in a large number of patients. To our knowledge, this is the largest study done specifically in NS patients with stroke. The results provide important information to clarify the pattern and outcome of stroke in patients with NS.

## Methods

### Ethical standards

The procedure of this clinical study was performed under a protocol approved by the Medical Ethics Committee of CG Memorial Hospital, Taipei, Taiwan in accord with the Helsinki Declaration of 1975 (the IRB approval number: 202001012B0). As all data were anonymized from existing databases and results were presented in aggregate, the requirement for informed consent was waived. The no requirement of informed consent was approved by the Medical Ethics Committee of CG Memorial Hospital, Taipei, Taiwan.

### Data source and collection

The CG Medical Foundation, which consists of branches of CG Memorial Hospital including two medical centers and two regional and three district hospitals, is the largest medical system in Taiwan [17]. The CG Medical Foundation has 10,070 beds, with admission of more than 280,000 patients each year [18]. All branches of CG Memorial Hospital apply electronic medical records. The CG Research Database is a de-identified database comprised of multi-institutional standardized electronic medical records, dating back to 2000, from the CG Medical Foundation. It contains demographic data, clinical diagnosis, medical records, laboratory data, radiological images, and reports [17].

### Study population

The study subjects were selected based on the CG Research Database from January 1, 2001, to December 31, 2017. The accuracy of the diagnoses of NS and stroke of the subjects was confirmed by the ICD-9-CM or ICD-10-CM codes combined with the records of discharge summary. We only recruited subjects with NS who were admitted for acute IS or nontraumatic ICH.

### Clinical assessments of IS

The IS subtypes were grouped by the Trial of ORG 10,172 in Acute Stroke Treatment classification system [19], and the clinical subtypes of IS were grouped by the Oxfordshire Community Stroke Project classification system [20]. Comorbidities, vascular risk factors, and clinical course were identified after an in-depth review of the medical records by three board-certified neurologists (Chang KH, Chang CW and Huang WY). Acute complications during admission were recorded. Laboratory tests including complete blood cell count, blood chemistry and brain imaging studies, coagulation testing, carotid duplex, and transthoracic echocardiography were recorded. The primary end point was 30-day mortality, and every cause of death was reviewed.

### Clinical assessments of ICH

The ICH subtypes were recorded, which included intracerebral hemorrhage (IH), subarachnoid hemorrhage (SAH), subdural hemorrhage, and arteriovenous malformation with hemorrhage. Comorbidities, vascular risk factors, clinical course, and laboratory tests were identified after an in-depth review of the medical records by three board-certified neurologists (Chang KH, Chang CW and Huang WY). The severities of IH and SAH were evaluated by the IH score [21] and Hunt and Hess scale, respectively. The primary end point was 30-day mortality, and every cause of death was reviewed.

### Statistical analysis

Continuous variables with normal distribution were expressed as mean ± standard deviation, whereas those that were not normally distributed were expressed as median (interquartile range). Categorical variables were expressed as a number (percentage) [22]. We used the “mean substitution” method to handle the missing laboratory data [23]. Different groups (IS and ICH) were compared using the chi-square (categorical variables) or Mann-Whitney *U* (continuous variables that were not normally distributed) or Student’s *t*-test (continuous variables with normal distribution) [22]. The characteristics and clinical course of the patients with IS classified by the Trial of ORG 10,172 in Acute Stroke Treatment classification system and patients with ICH were analyzed using descriptive statistics, and the group differences were assessed using one-way ANOVA (continuous variables with normal distribution) or the Kruskal-Wallis (continuous variables that were not normally distributed) or chi-square (categorical variables) test [22]. The Kaplan-Meier survival analysis was used to estimate the cumulative overall survival for IS and ICH, and the group differences were assessed using the log-rank test [24]. The Cox proportional hazards model was used to determine the significance of each variable in predicting the 30-day mortality. A univariate Cox model, assessing all previously identified variables, was used to measure the hazard ratio for mortality. All variables with *P* < 0.1 in the univariate Cox regression analysis entered a stepwise, backward multivariate Cox regression analysis. A backward, stepwise multivariate Cox regression model was also used to identify the risk factors for the 30-day mortality, and the variables with *P* < 0.05 remained for the final model [24]. All statistical analyses were performed with IBM SPSS statistics 19 for Windows.

## Results

### Patient selection

We recruited in hospitalization records of patients in CG Research Databases from January 1, 2001, to December 31, 2017, using the NS-related ICD-9-CM or ICD-10-CM codes, and 3352 hospitalization records were identified. The selected hospitalized records (include the discharge summary and laboratory data) were screened by the board-certified nephrologist (Chen KH) and neurologist (Huang WY, Chang CC and Chang KH). After excluding 1503 patients that did not present with NS, and 1559 patients that are not admitted with the diagnosis of acute stroke, we obtained 290 patients, including 233 patients with IS and 57 patients with ICH, for further analysis (Supplementary Figure 1). The percentage of missing laboratory data ranged from 0 to 15.8 % (Supplementary Table 1).

### Demographic characteristics among patients with any stroke

A total of 290 patients with NS who had been hospitalized for acute IS (*n* = 233, 80.3 %) or ICH (*n* = 57, 19.7 %) were enrolled in the study. Patient characteristics are presented in Table 1 and Supplementary Table 2. The median age was 60 (52–70) years. The prevalence rates of hyperlipidemia (63.9 %), hyperuricemia (17.2 %), and smoking (9.4 %) in patients with IS were significantly higher than in those with ICH (hyperlipidemia, 29.8 %, *P* < 0.001; hyperuricemia, 7 %, *P* = 0.037; smoking, 1.8 %, *P* = 0.037). In laboratory data, patients with ICH demonstrated higher white blood cell (WBC) count (8.92 × 10^9^/L) and high-sensitivity C-reactive protein (hs-CRP) level (144.10 nmol/L) compared with those with IS (WBC count, 7.80 × 10^9^/L, *P* = 0.031; hs-CRP, 33.42 nmol/L, *P* = 0.001). By contrast, the cholesterol (4.84 mmol/L), triglyceride (1.28 mmol/L) and albumin (18 g/L) levels were significantly lower in patients with ICH compared with those in patient with IS (cholesterol, 5.74 mmol/L, *P* = 0.004; triglyceride, 1.60 mmol/L, *P* < 0.001; albumin, 24 g/L, *P* = 0.009).

**Table 1:** Demographic features of nephrotic syndrome patients with stroke

	Ischemic stroke (*n* = 233)	Intracranial hemorrhage (*n* = 57)	Odds ratio (95% confidence interval)	*P* value
Age (years)	61 (52–71)	57 (52–69)		0.446
Male (%)	145 (62.2)	35 (61.4)	1.04 (0.57–1.88)	0.512
Risk factors				
Hypertension (%)	195 (83.7)	47 (82.5)	1.09 (0.51–2.35)	0.479
Diabetes mellitus (%)	152 (65.2)	30 (52.6)	1.69 (0.94–3.03)	0.055
Hyperlipidemia (%)	149 (63.9)	17 (29.8)	4.17 (2.23–7.82)	< 0.001†
Coronary artery disease (%)	35 (15)	8 (14)	1.08 (0.47–2.48)	0.520
Congestive heart failure (%)	32 (13.7)	9 (15.8)	0.85 (0.38–1.90)	0.414
Atrial fibrillation (%)	24 (10.3)	4 (7)	1.52 (0.51–4.57)	0.320
Hyperuricemia (%)	40 (17.2)	4 (7)	2.75 (0.94–8.02)	0.037†
Old stroke (%)	77 (33)	17 (29.8)	1.16 (0.62–2.18)	0.383
Smoking (%)	22 (9.4)	1 (1.8)	5.84 (0.77–44.26)	0.037†
Lab data				
White blood cells (×10^9^/L)	7.80 (6.21–9.25)	8.92 (6.95–11.02)		0.031*
High-sensitivity C-reactive protein (nmol/L)	33.42 (14.48–120.10)	144.10 (56.29–330.76)		0.001*
Total cholesterol (mmol/L)	5.74 (4.24–6.85)	4.84 (3.39–6.31)		0.004*
Triglyceride (mmol/L)	1.60 (1.07–2.73)	1.28 (0.95–1.71)		< 0.001*
Albumin (g/L)	24 (11–31)	18 (15–29)		0.009*

Data are presented as median (interquartile range) or absolute numbers (percentage)

*P < 0.05, Mann-Whitney *U* test; † *P* < 0.05, Chi-square test

### Demographic characteristics and clinical course among patients with IS

Of 233 patients with NS who had been hospitalized for acute IS, 43 (18.5 %) had large-artery atherosclerosis; 23 (9.9 %), cardioembolism; 117 (50.2 %), small-artery occlusion; 11 (4.7 %), stroke of other determined etiology; and 39 (16.7 %), stroke of undetermined etiology. Patient characteristics and clinical course are summarized in Table 2 and Supplementary Table 3. The prevalence of atrial fibrillation was higher in the cardioembolism group (87.0 %, *P* < 0.001); the prevalence of diabetes mellitus was higher in the small-artery occlusion (80.3 %) and large-artery atherosclerosis (69.8 %) groups (*P* < 0.001); the prevalence of modified Rankin scale score ≧ 3 upon discharge was higher in the cardioembolism (78.3 %), stroke of undetermined etiology (76.9 %), and large-artery atherosclerosis (76.7 %) groups (*P* = 0.002). Of the female IS patients (total 88 patients), the 30-day mortality rate was 2.3 % (2 patients died); whereas of the male IS patient (total 145 patients), the 30-day mortality was 4.1 % (6 patients died). There was no significant sex difference in patients with IS (*P* = 0.361, odds ratio for male = 1.86, 95 % confidence interval 0.37–9.41).

**Table 2:** Demographic features and clinical courses of nephrotic syndrome patients with ischemic stroke

Subtypes	All ischemic stroke (*n* = 233)					*P* value
	Large-artery atherosclerosis (*n* = 43)	Cardioembolism (*n* = 23)	Small-artery occlusion (*n* = 117)	Stroke of other determined etiology (*n* = 11)	Stroke of undetermined etiology (*n* = 39)	
Age (years)	58 (50–73)	68 (61–76)	60 (54–69)	35 (29–42)	63 (53–71)	< 0.001*
Male (%)	25 (58.1)	16 (69.6)	74 (63.2)	6 (54.5)	24 (61.5)	0.883
Risk factors (%)						
Hypertension (%)	38 (88.4)	21 (91.3)	100 (85.4)	7 (63.6)	29 (74.4)	0.105
Diabetes mellitus (%)	30 (69.8)	7 (30.4)^†^	94 (80.3)^†^	1 (9.1)^†^	20 (51.3)	< 0.001†
Hyperlipidemia (%)	30 (69.8)	7(30.4)^†^	81 (69.2)	6 (54.5)	25 (64.1)	0.008†
Coronary artery disease (%)	36 (83.7)	6 (26.1)	19 (16.2)	0 (0)	7 (17.9)	0.151
Congestive heart failure (%)	8 (18.6)	6 (26.1)	12 (10.3)	1 (9.1)	5 (12.8)	0.263
Atrial fibrillation (%)	0 (0)	20 (87.0)^†^	2 (1.7)^†^	0 (0)	1 (2.6)	< 0.001†
Hyperuricemia (%)	10 (23.3)	8 (34.8)	13 (11.1)	3 (27.3)	6 (15.4)	0.040†
Old stroke (%)	18 (41.9)	6 (26.1)	36 (30.8)	3 (27.3)	14 (35.9)	0.627
Smoking (%)	4 (9.3)	0 (0)	15 (12.8)	0 (0)	3 (7.7)	0.263
Mean length of stay in the acute medicine ward(day)	14 (10–21)	19 (9–29)	9 (6–13)	12 (6–20)	13 (9–19)	< 0.001*
Complication at admission						
Pneumonia (%)	12 (27.9)	5 (21.7)	11 (9.4)	1 (9.1)	11 (28.2)	0.013†
Gastrointestinal bleeding (%)	5 (11.6)	2 (8.7)	5 (4.3)	1 (9.1)	8 (20.5)	0.043†
Urinary tract infection (%)	5 (11.6)	6 (26.1)	12 (10.3)	1 (9.1)	5 (12.8)	< 0.001†
Modified Rankin scale score ≧ 3 upon discharge (%)	33 (76.7)	18 (78.3)	67 (57.3)	3 (27.3)	30 (76.9%)	0.002†
Death within 30 days (%)	4 (9.3)	1 (4.3)	2 (1.7)	0 (0)	1 (2.6)	0.195

Data are presented as median (interquartile range) or absolute numbers (percentage)

*P < 0.05, Kruskal-Wallis test; †*P* < 0.05, Chi-square test

### Demographic characteristics and clinical course among patients with ICH

Of 57 patients with NS who had been hospitalized for ICH, 45 (78.9 %) had IH; 4 (7.0 %), SAH; 7 (12.3 %), subdural hemorrhage; and 1 (1.8 %), arteriovenous malformation with bleeding. Patient characteristics and outcomes are presented in Table 3 and Supplementary Table 4. The 30-day mortality was the highest in the SAH group (75 %, *P* = 0.036). The prevalence of modified Rankin scale score ≧ 3upon discharge was higher in the IH group (97.8 %, *P* < 0.001).

**Table 3:** Demographic features and clinical courses of nephrotic syndrome patients with intracranial hemorrhage

Types of hemorrhage	Any intracranial hemorrhage (*n* = 57)				*P* value
	Intracerebral hemorrhage (*n* = 45)	Subarachnoid hemorrhage (*n* = 4)	Subdural hemorrhage (*n* = 7)	Arteriovenous malformation (*n* = 1)	
Age (years)	57 (52–63)	59 (38–76)	70 (57–81)	29	0.064
Male (%)	27 (60)	2 (50)	5 (71.4)	1 (100)	0.757
Risk factors					
Hypertension (%)	39 (86.7)	3 (75)	5 (71.4)	0 (0)	0.112
Diabetes mellitus (%)	25 (55.6)	1 (25)	4 (57.1)	0 (0)	0.467
Hyperlipidemia (%)	14 (31.1)	2 (50)	1 (14.3)	0 (0)	0.563
Coronary artery disease (%)	7 (15.6)	0 (0)	1 (14.3)	0 (0)	0.825
Congestive heart failure (%)	8 (17.8)	0 (0)	1 (14.3)	0 (0)	0.781
Atrial fibrillation (%)	4 (8.9)	0 (0)	0 (0)	0 (0)	0.766
Hyperuricemia (%)	3 (6.7)	1 (25)	0 (0)	0 (0)	0.458
Smoking (%)	1 (2.2)	0 (0)	0 (0)	0 (0)	0.965
Mean length of stay in the acute medicine ward (day)	15 (11–28)	22 (18–48)	9 (8–14)	16	0.154
Modified Rankin Scale score ≧ 3 upon discharge (%)	44 (97.8)	3 (75)	4 (57.1)	0 (0)	< 0.001*
Death within 30 days (%)	10 (22.2)	3 (75)	0 (0)	0 (0)	0.036*
Score of hemorrhage severity (%)	Intracerebral hemorrhage score: All: 2 (1–3) Death: 3 (3–4) Non-death:1 (1–3)	Hunt and Hess scale score: All: 4 (2–4) Death: 4 (4–4) Non-death: 2 (2–2)			

Data are presented as median (interquartile range) or absolute numbers (percentage)

*P < 0.05, Chi-square test

### Survival analysis of mortality in patients with any stroke

**Fig. 1:**
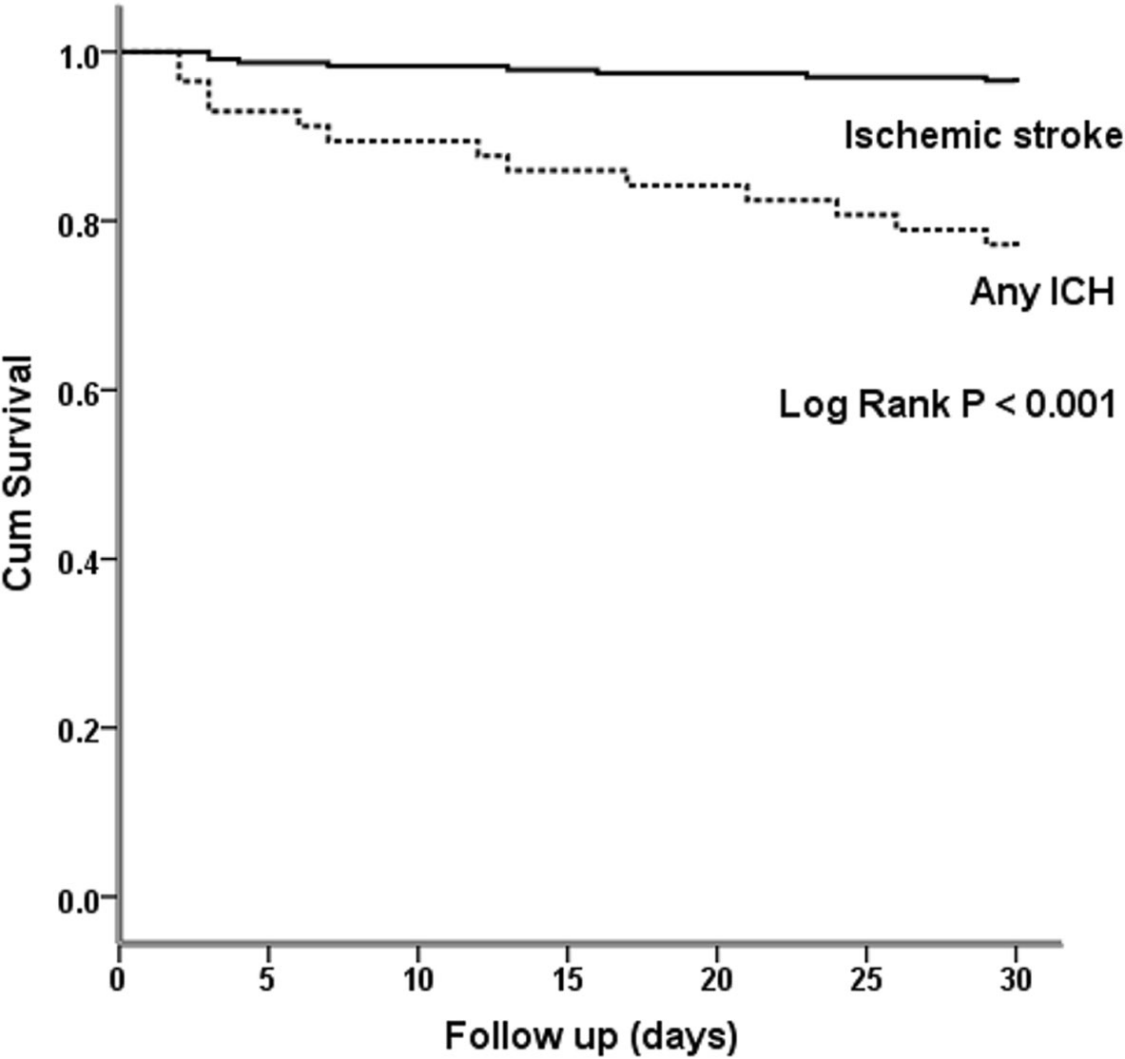
Kaplan–Meier estimates of patient survival (all-cause mortality) during the 30-day study period. Symbols are as follows: ∙∙∙∙, any intracranial hemorrhage (ICH); and —, ischemic stroke. Log-rank test, *P* < 0.001

During the 30-day observation period, 21 patients died (21/290 = 7.2 %), including 8 (3.4 %) patients with IS and 13 (22.8 %) patients with ICH. The causes of patients’ death in IS included sepsis (3 patients), pneumonia (2 patients), large infarction with brainstem compression (1 patient), acute myocardial infarction (1 patient), and cardiac arrhythmia (1 patient). The causes of death in patients with ICH were brainstem dysfunction (10 patients) and sepsis (3 patients). The Kaplan–Meier analysis showed that patients with ICH had a higher mortality rate compared with those with IS (log-rank test, *P* < 0.001, Fig. 1).

### Determinants for mortality in patients with any stroke

The univariate Cox regression indicated that low lipid level, coronary artery disease (CAD), congestive heart failure, pneumonia, ICH, and IS presented as total anterior circulation syndrome (TACS) were potential risk factors (*P* < 0.1) for the 30-day all-cause mortality. The multivariate Cox proportional hazards model still showed CAD, ICH, and TACS as significant risk factors for the 30-day all-cause mortality after adjusting for these variables (Table 4 and Supplementary Table 5).

**Table 4:** Cox regression analysis of patient survival during the 30-day period, intracranial hemorrhage vs. ischemic stroke

	Univariate Cox regression		Multivariate Cox regression	
	HR (95% CI)	*P* value	HR (95% CI)	*P* value
Hyperlipidemia	0.22 (0.08–0.60)	0.003*		
Coronary artery disease	3.12 (1.26–7.72)	0.014*	4.19 (1.67–10.46)	0.002†
Congestive heart failure	2.58 (1.00–6.65)	0.050*		
Pneumonia	3.66 (1.54–8.68)	0.003*		
Intracranial hemorrhage	7.33 (3.04–17.70)	< 0.001*	10.68 (3.31–34.47)	< 0.001†
Total anterior circulation syndrome	4.82 (1.62–14.34)	0.005*	17.65 (4.37–71.23)	< 0.001†

*HR* Indicates hazard ratio, *CI* Confidence interval

*P < 0.1 for the univariate Cox regression, and †*P* < 0.05 for the multivariate Cox regression

### Determinants of mortality in patients with IS

The univariate Cox regression revealed that CAD, congestive heart failure, TACS, pneumonia, and elevated hs-CRP level were positively associated, while hyperlipidemia was negatively associated with 30-day mortality among NS patients with IS. After adjusting for these potential risk factors (*P* < 0.1) in a backward multivariate Cox proportional hazards model, only CAD was positively associated with 30-day mortality in NS patients with IS (Table 5 and Supplementary Table 6).

**Table 5:** Cox regression analysis of patient survival during the 30-day period in nephrotic syndrome patients with stroke

Variables	Univariate Cox regression, HR (95% CI)	*P* value	Multivariate Cox regression, HR (95% CI)	*P* value
**Ischemic stroke**				
Hyperlipidemia	0.18 (0.04–0.91)	0.037*		
Coronary artery disease	3.50 (0.84–14.64)	0.086*	24.58 (1.48–408.90)	0.026†
Congestive heart failure	3.83 (0.92–16.02)	0.066*		
Total anterior circulation syndrome	16.41 (4.10–65.72)	< 0.001*		
Pneumonia	8.34 (1.99–34.92)	0.004*		
High-sensitivity C-reactive protein	1.04 (1.02–1.06)	< 0.001*		
**Intracranial hemorrhage**				
Male	0.37 (0.12–1.12)	0.078*		
Coronary artery disease	3.93 (1.20–12.85)	0.023*	5.49 (1.54–19.56)	0.009†
Subarachnoid hemorrhage	4.23 (1.15–15.52)	0.030*	6.32 (1.57–25.53)	0.010†

*HR* Indicates hazard ratio, *CI* Confidence interval

*P < 0.1 for the univariate Cox regression, and †*P* < 0.05 for the multivariate Cox regression

### Determinants of mortality in patients with ICH

The univariate Cox regression revealed that CAD and SAH were positively associated, while male sex was negatively associated with 30-day mortality among NS patients with IS. After adjusting for these potential risk factors (*P* < 0.1) backward multivariate Cox proportional hazards model, CAD and SAH were positively associated with 30-day mortality in NS patients with ICH (Table 5 and Supplementary Table 7).

## Discussion

As previous limited studies suggest that the incidence rates of IS and ICH both increase in patients with NS compared with those of the general population, it is important to identify the factors associated with these two types of stroke in NS. To our knowledge, this study is the largest study done specifically in NS patients with stroke. Our results demonstrated that the prevalence rates of hyperlipidemia, hyperuricemia, and smoking were significantly higher in patients with IS compared with those in patients with ICH. Lines of evidence suggest that lower cholesterol level increases the risk of ICH [25, 26]. However, the association between cholesterol and risk of IS remains undetermined. A number of reports suggest that cholesterol, high-density lipoprotein cholesterol, and triglycerides are not significantly associated with IS risk [27, 28]. By contrast, one large previous cohort study of healthy women suggests that cholesterol and low-density lipoprotein cholesterol are significantly associated with increased risk of IS [29]. Interestingly, a prospective study demonstrates a positive association of cholesterol with the risk of IS in men, and an inverse association of cholesterol with IH in women [30]. In NS patients with IS, the prevalence of hyperlipidemia was higher in large-artery atherosclerosis and small-artery occlusion when compared to cardioembolism. In NS patients with ICH, the association between hyperlipidemia and the subtypes of ICH was not significant. Other risk factors for stroke in patients with NS, including the higher prevalence of smoking and hyperuricemia in patients with IS, are consistent with those of the global populations [31–35].

Comparisons of the risk factors between IS and ICH in general population had been performed in previous studies, but the results remained inconclusive. Factors favoring IS versus ICH which have been described in previous studies included DM [36–38], AF [37–39], previous myocardial infarction [37, 38], previous stroke [37], intermittent arterial claudication [37, 39], obesity [36], hypertension [39], and hyperlipidemia [40]. On the other hand, factors favoring ICH versus IS included smoking [37], alcohol consumption [37, 38], and hypertension [38, 40]. In patients with NS, the prevalence of hyperlipidemia, hyperuricemia, and smoking were significantly higher in IS when compared to ICH. Reduction of cholesterol and uric acid levels, and cassation of cigarette smoking would be important to prevent IS in patients with NS.

Our study also finds higher WBC count and hs-CRP levels as well as lower albumin levels in patients with ICH compared with those in patients with IS. Infection-unrelated elevation of WBC count can be observed at the early phase after ICH [41–43]. High WBC counts may be also associated with IS in patients with CAD [44]. Higher hs-CRP level predicts further cerebral ischemic events in patients with IS [45] and early hematoma growth and neurological worsening in patients with IH [46]. It has been shown that patients with large stroke volume have high WBC count and CRP levels in the acute phase of stroke [47]. Consistent with a previous finding [34, 35], we demonstrated that the albumin levels in patients with IS are higher than in those with ICH.

Small-artery occlusion (50.2 %) is the leading cause of IS in patients with NS. The lower mortality rate in patients with small-artery occlusion also significantly contributes to the relatively low mortality in all patients with IS. Atrial fibrillation is the major cause of cardioembolic stroke [48] and frequently seen in cardioembolism (87 %) in NS patients with IS. Systemic lupus erythematosus, which is associated with both IS and ICH [49], is frequently seen in stroke of other determined etiology (54.5 %) in NS patients with IS.

Our results show that the mean duration of acute ward stay, percentage of dependent functional outcome (modified Rankin scale score ≧ 3), and 30-day mortality are higher in patients with ICH (subtypes: IH and SAH) than in those with IS. These findings suggest that the severity of ICH in patients with NS is greater, accompanied with worse outcome, than in patients with IS. The clinical characteristics and outcome of patients with NS and ICH were seldom reported. Only one study demonstrates 15 patients with ICH and NS [11]. In this study, more than half of the patients presented with comatose status, and a majority of NS patients with ICH had a poor prognosis [11]. Our study, which recruited 57 patients with ICH, demonstrates that SAH and IH had higher mortality than other ICH subtypes (75% and 22.2 %, respectively). In general population, the mortality rates of SAH divided by the Hunt and Hess scale score were 3 % for Hunt and Hess grade 1 or 2, 9 % for grade 3, 24 % for grade 4, and 71 % for grade 5 [50]. In our study, all patients with Hunt and Hess grade 4 died, suggesting the worse survival in NS patients with SAH. The multivariate Cox regression analysis further confirms the correlation of SAH with 30-day mortality in NS patients with ICH.

Our results show that CAD, ICH and IS presented as TACS were associated with the 30-day mortality in NS patients with any type of stroke. The association between carotid atherosclerosis and CAD has been well established since atherosclerosis is regarded as a systemic disease [51]. The percentage of 30-day mortality in our study is 7.2 %, which is similar to a previous study with a small number of patients [11]. The multivariate Cox regression analysis further confirms the correlation of the presence of CAD with either IS or ICH. It is not surprising that TACS, which is usually involved in a large volume of infarction, is associated with poor functional outcome and high all-cause mortality [52].

This study has several limitations. First, there may be a selection bias toward more patients with severe stroke or NS because CG Memorial Hospitals are the largest referral hospitals in Taiwan and have larger proportions of patients with higher disease severity. Second, the data of patients treated in other hospitals were not available in the CG Research Database. Therefore, the dataset may not be able to represent the whole disease group of the overall Taiwanese population. Third, this population-based study has a retrospective design, which may have introduced some confounding factors that could influence the analysis. Fourth, we did not compare IS versus non-IS or ICH versus non-ICH in patients with NS, and may not identify the risk factors for both stroke types. Fifth, the outcome events per predictor variable in the multivariate Cox regression model of our study was relatively stretched. However, one previous study suggested that the rule of ten events per predictor variable in Cox regression might be safely relaxed but should be interpreted with caution [53]. Sixth, the details of NS (e.g., staging of chronic kidney disease, immunosuppressive therapies, blood pressure medications, and treatment response, etc.), were not available in most patients of the CG Research Databases.

## Conclusions

NS patients with acute ICH are associated with significantly higher 30-day all-cause mortality compared with those with acute IS. Therefore, the use of antithrombotic agents for IS prevention should be cautious in patients with numerous cerebral microbleeds or cerebral aneurysms with high bleeding risk since the 30-day mortality is high in these patients if ICH occurs.

## Supplementary Information

**Additional file 1: Supplementary Figure 1.** Flow of study selection. NS indicates nephrotic syndrome; IS, ischemic stroke; ICH, intracranial hemorrhage. **Supplementary Table 1.** The numbers of missing laboratory data. **Supplementary Table 2.** Demographic features of nephrotic syndrome patients with stroke (additional data). **Supplementary Table 3.** Demographic features and clinical courses of nephrotic syndrome patients with ischemic stroke (additional data). **Supplementary Table 4.** Demographic features and clinical courses of nephrotic syndrome patients with intracranial hemorrhage (additional data). **Supplementary Table 5.** Cox regression analysis of patient survival during the 30-day period, intracranial hemorrhage vs. ischemic stroke (detailed items). **Supplementary Table 6.** Cox regression analysis of patient survival during the 30-day period in nephrotic syndrome patients with ischemic stroke (detailed items). **Supplementary Table 7.** Cox regression analysis of patient survival during the 30-day period in nephrotic syndrome patients with intracranial hemorrhage (detailed items).

## Data Availability

The dataset analyzed during the current study are available from the corresponding author on reasonable request.

## Abbreviations

NS: Nephrotic syndrome
IS: Ischemic stroke
ICH: Intracranial hemorrhage
CG: Chang Gung
IH: Intracerebral hemorrhage
SAH: Subarachnoid hemorrhage
WBC: White blood cell
hs-CRP: High-sensitivity C-reactive protein
CAD: Coronary artery disease
TACS: Total anterior circulation syndrome
