# Supplementary material for: Characteristics of ischemic stroke and intracranial hemorrhage in patients with nephrotic syndrome
Source: BMC Nephrol. 2021 Jun 5;22:213. doi: 10.1186/s12882-021-02415-w (PMC8178873; doi:10.1186/s12882-021-02415-w)
Supplement: Supplementary file 1 — Additional file 1: Supplementary Figure 1. Flow of study selection. NS indicates nephrotic syndrome; IS, ischemic stroke; ICH, intracranial hemorrhage. Supplementary Table 1. The numbers of missing laboratory data. Supplementary Table 2. Demographic features of nephrotic syndrome patients with stroke (additional data). Supplementary Table 3. Demographic features and clinical courses of nephrotic syndrome patients with ischemic stroke (additional data). Supplementary Table 4. Demographic features and clinical courses of nephrotic syndrome patients with intracranial hemorrhage (additional data). Supplementary Table 5. Cox regression analysis of patient survival during the 30-day period, intracranial hemorrhage vs. ischemic stroke (detailed items). Supplementary Table 6. Cox regression analysis of patient survival during the 30-day period in nephrotic syndrome patients with ischemic stroke (detailed items). Supplementary Table 7. Cox regression analysis of patient survival during the 30-day period in nephrotic syndrome patients with intracranial hemorrhage (detailed items). [file 12882_2021_2415_MOESM1_ESM.pdf]

TITLE PAGE

Full title:

Characteristics of Ischemic Stroke and Intracranial Hemorrhage in Patients with Nephrotic Syndrome

Authors' name:

Wen-Yi Huang, Chun-Wei Chang, Chiung-Mei Chen, Kuan-Hsing Chen, MD, Chien-Hung Chang, Hsiu-Chuan Wu, Kuo-Hsuan Chang*

Additional File 1

Supplementary Figure 1. Flow of study selection. NS indicates nephrotic syndrome; IS, ischemic stroke; ICH, intracranial hemorrhage.

Supplementary Table 1. The numbers of missing laboratory data.

Supplementary Table 2. Demographic features of nephrotic syndrome patients with stroke (additional data).

Supplementary Table 3. Demographic features and clinical courses of nephrotic syndrome patients with ischemic stroke (additional data).

Supplementary Table 4. Demographic features and clinical courses of nephrotic syndrome patients with intracranial hemorrhage (additional data).

Supplementary Table 5. Cox regression analysis of patient survival during the 30-day period, intracranial hemorrhage vs. ischemic stroke (detailed items).

Supplementary Table 6. Cox regression analysis of patient survival during the 30-day period in nephrotic syndrome patients with ischemic stroke (detailed items).

Supplementary Table 7. Cox regression analysis of patient survival during the 30-day period in nephrotic syndrome patients with intracranial hemorrhage (detailed items).

Supplementary Figure 1. Flow of study selection. NS indicates nephrotic syndrome; IS, ischemic stroke; ICH, intracranial hemorrhage.

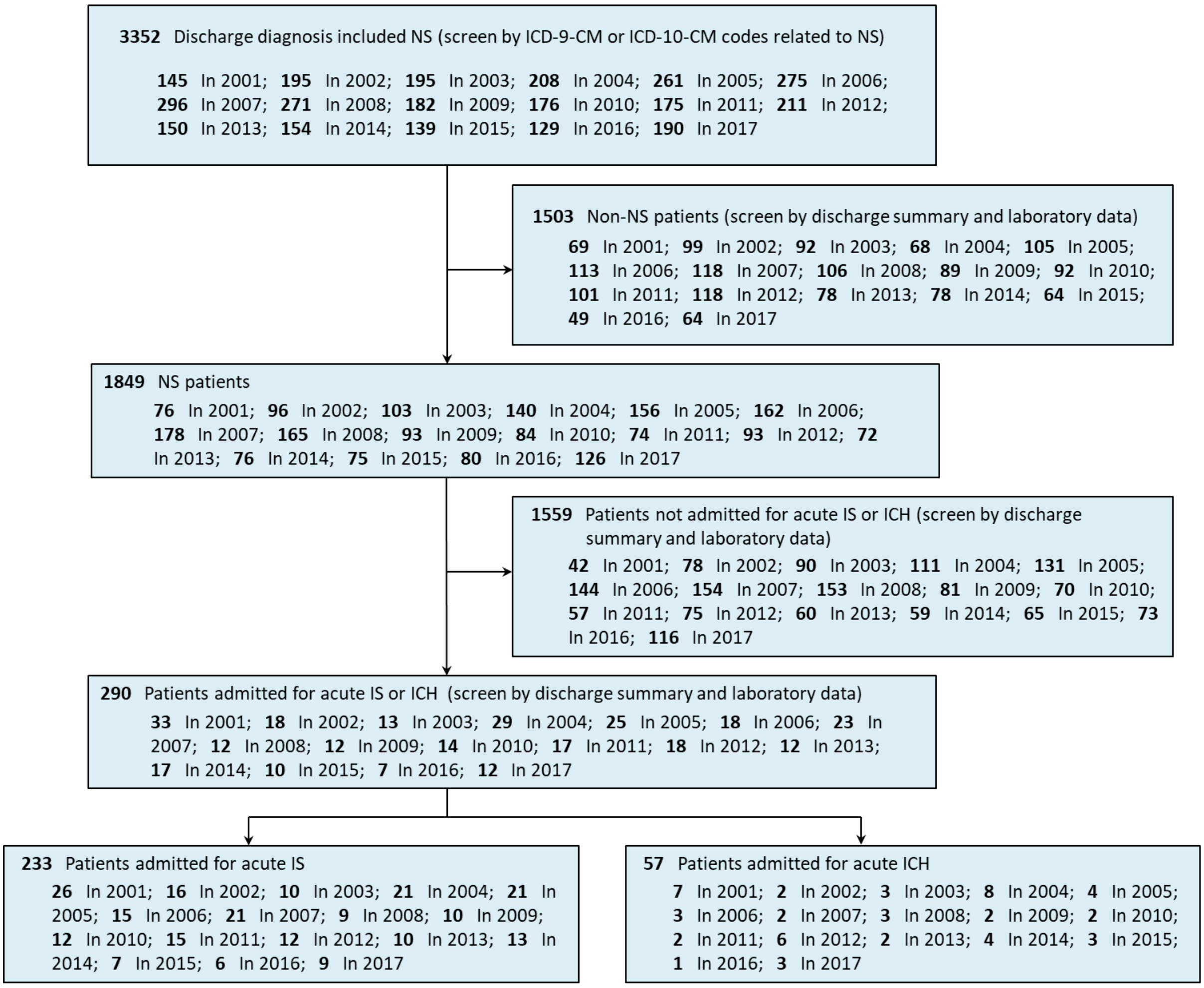

Supplementary Table 1. The numbers of missing laboratory data.

	The number of missing data, n (%)	
	Ischemic stroke (n=233)	Any intracranial hemorrhage (n=57)
White blood cells	0 (0)	0 (0)
Hemoglobin	0 (0)	0 (0)
Platelet	0 (0)	0 (0)
INR	10 (4.3)	2 (3.5)
APTT/NC	10 (4.3)	2 (3.5)
High-sensitivity C-reactive protein	21 (9.0)	5 (8.8)
ESR	24 (10.3)	7 (12.2)
Total cholesterol	10 (4.3)	2 (3.5)
Triglyceride	11 (4.7)	2 (3.5)
LDL	17 (7.6)	4 (7.0)
HDL	17 (7.6)	4 (7.0)
Albumin	13 (5.6)	3 (5.3)
Total protein	18 (7.7)	5 (8.8)
Sugar	29 (12.4)	9 (15.8)
Glycohemoglobin	21 (9)	6 (10.5)
BUN	10 (4.3)	2 (3.5)
Creatinine	0 (0)	0 (0)
AST	17 (7.6)	5 (8.8)
ALT	16 (6.9)	5 (8.8)
Uric acid	26 (11.2)	8 (14.0)
Sodium	14 (6.0)	6 (10.5)
Potassium	11 (4.7)	5 (8.8)
Calcium	25 (10.7)	7 (12.3)
Phosphate	29 (12.4)	8 (14.0)

INR indicates international normalized ratio; APTT/NC, activated partial thromboplastin time/normal control; ESR, erythrocyte sedimentation rate; LDL, low-density lipoprotein; HDL, high-density lipoprotein; BUN, blood urea nitrogen; AST, aspartate transaminase; ALT, alanine aminotransferase; ANA, antinuclear antibody; VDRL, the venereal disease research laboratory test.

Supplementary Table 2. Demographic features of nephrotic syndrome patients with stroke (additional data).

	Ischemic stroke (n=233)	Any intracranial hemorrhage (n=57)	Odds ratio (95% confidence interval)	*P* value
Height (cm)	163 (157-168)	165 (158-167)		0.831
Body weight (kg)	63 (58-69)	62 (55-70)		0.828
BMI (kg/m^2)	24.6 (22.7-26.3)	21.1 (19.3-24.8)		0.357
SBP (mmHg)	169 (150-190)	167 (132-192)		0.176
DBP (mmHg)	90 (78-96)	87 (80-102)		0.889
Other diseases				
Chronic obstructive lung disease (%)	12 (9.9)	1 (1.8)	3.04 (0.39-23.9)	0.236
Systemic lupus erythematosis (%)	6 (2.8)	1 (1.8)	1.48 (0.18-12.5)	0.585
End-stage renal disease with dialysis (%)	35 (15)	11 (19.3)	0.74 (0.35-1.56)	0.272
Lab data				
Hemoglobin (mmol/L)	7.39 (6.45-8.25)	7.23 (6.14-8.13)		0.312
Platelet (×10^9/L)	214 (170-269)	213 (161-258)		0.801
INR	0.99 (0.90-1.09)	1.09 (0.98-1.20)		0.371
APTT/NC	0.99 (0.92-1.06)	0.99 (0.89-1.04)		0.574
Sugar (mmol/L)	7.9 (6.3-8.6)	6.8 (6.5-8.1)		0.220
Glycohemoglobin (%)	7.7 (6.0-9.4)	7.63 (5.9-8.8)		0.372
ESR (mm/hr)	34.5 (18.3-70.3)	25.5 (13.3-64.8)		0.545
BUN (mmol/L)	8.31 (6.4-19-64)	8.25 (5.07-18.64)		0.063
Creatinine (μmol/L)	155.47 (106.12-336.21)	153.30 (83.11-308.59)		0.941
AST (μkat/L)	0.35 (0.25-0.58)	0.31 (0.22-0.52)		0.443
ALT (μkat/L)	0.32 (0.23-0.61)	0.34 (0.21-0.54)		0.601

LDL (mmol/L)	3.91 (3.11-4.66)	3.88 (2.98-4.69)		0.834
HDL (mmol/L)	1.06 (0.92-1.31)	1.13 (0.93-1.29)		0.394
Uric acid (μmol/L)	392.5 (303.3-472.3)	386.6 (327.1-469.9)		0.896
Sodium (mmol/L)	138.9 (132.1-146.7)	134.5 (131.5- 145.9)		0.262
Potassium (mmol/L)	4.1 (3.5-4.8)	3.9 (3.3-4.7)		0.361
Calcium (mmol/L)	2.14 (2.09-2.72)	2.16 (2.02-2.64)		0.705
Phosphate (mmol/L)	1.17 (1.13-1.45)	1.15 (1.12-1.45)		0.453
Total protein (g/L)	52 (32-59)	42 (35-50)		0.460
ANA (%)	3 (1.3)	0 (0)	1.01 (1.00-1.03)	0.517
VDRL (%)	4 (1.7)	0 (0)	1.02 (1.00-1.04)	0.415

BMI indicates body mass index; SBP, systolic blood pressure; DBP, diastolic blood pressure; INR, international normalized ratio; APTT/NC, activated partial thromboplastin time/normal control; ESR, erythrocyte sedimentation rate; BUN, blood urea nitrogen; AST, aspartate transaminase; ALT, alanine aminotransferase; LDL, low-density lipoprotein; HDL, high-density lipoprotein; ANA, antinuclear antibody; VDRL, the venereal disease research laboratory test.

Data are presented as median (interquartile range) or absolute numbers (percentage).
*P < 0.05, Mann-Whitney U test; † P < 0.05, Chi-square test.

Supplementary Table 3. Demographic features and clinical courses of nephrotic syndrome patients with ischemic stroke (additional data).

	All ischemic stroke (n=233)					
Subtypes	Large-artery atherosclerosis (n=43)	Cardioembolism (n=23)	Small-artery occlusion (n=117)	Stroke of other determined etiology (n=11)	Stroke of undetermined etiology (n=39)	*P* value
Height (cm)	162 (158-169)	163 (161-168)	162 (158-169)	166 (158-169)	164 (159-169)	0.654
Body weight (kg)	61 (55-68)	67 (62-71)	64. (55-69)	57 (53-61)	59 (52-64)	0.122
BMI (kg/m^2)	23.8 (22.3-28.1)	25.0 (23.9-25.6)	24.8 (23.2-26.4)	22.1 (19.2-23.1)	23.3 (22.2-25.9)	0.065
SBP (mmHg)	175 (155-181)	160 (130-185)	176 (150-190)	160 (135-171)	170 (155-191)	0.439
DBP (mmHg)	95 (85-100)	88 (78-95)	90 (76-97)	86 (76-96)	90 (75-101)	0.922
Other diseases						
Chronic obstructive lung disease (%)	5 (11.6)	4 (17.4)	2 (1.7)	0 (0)	1 (0.4)	0.005†
Systemic lupus erythematosis (%)	0 (0)	0 (0)	0 (0)	6 (54.5)†	0 (0)	<0.001†
End-stage renal disease with dialysis (%)	8 (18.6)	2 (8.7)	17 (14.5)	0 (0)	8 (20.5)	0.400
Clinical syndromes						
TACS (%)	8 (18.6)	6 (26.1)	0 (0)	0 (0)	1 (2.6)	<0.001†
PACS (%)	13 (30.2)	7 (30.4)	19 (16.2)	8 (72.7)	20 (51.3)	<0.001†
LACS (%)	8 (18.6)	4 (17.4)	71 (60.7)	0 (0)	7 (17.9)	<0.001†
POCS (%)	14 (32.6)	6 (26.1)	27 (23.1)	3 (27.3)	10 (25.6)	0.828

Lab data						
WBC (×10^9/L)	8.32 (6.21-10.25)	7.95 (5.99-9.21)	7.86 (5.76-9.11)	9.12 (6.07-10.11)	8.21 (5.65-9.54)	0.226
Hemoglobin (mmol/L)	7.38 (6.41-8.35)	7.82 (6.52-8.41)	7.45 (6.22-8.35)	7.63 (6.11-8.79)	7.14 (5.99-8.43)	0.442
Platelet (×10^9/L)	205 (145-330)	231 (160-310)	217 (140-289)	212 (152-320)	220 (155-310)	0.188
INR	0.91 (0.89-1.12)	1.25 (0.98-1.42)	1.10 (0.98-1.31	1.01 (0.91-1.15)	0.93 (0.89-1.19)	0.066
APTT/NC	1.02 (0.91-1.21)	1.71 (1.41-1.92)	1.11 (0.98-1.13)	1.03 (1.01-1.21)	1.51 (1.31-1.61)	0.068
Sugar (mmol/L)	8.4 (7.3-8.8)	6.7 (5.9-7.6)	8.0 (6.9-.8.5)	5.4 (4.9-6.1)	5.7 (5.1-6.4)	0.300
Glycohemoglobin (%)	8.62 (5.92-9.11)	6.83 (5.89-7.23)	8.30 (6.10-8.98)	6.35 (5.87-7.95)	7.61 (5.89-8.91)	0.057
Hs-CRP (nmol/L)	208.57 (44.41-331.52)	137.14 (37.14-241.91)	97.14 (32.14-139.71)	84.76 (24.21-119.25)	141.90 (33.42-221-57)	0.400
ESR (mm/hr)	57.4 (20.3-69.2)	29.2 (10.1-48.7)	43.8 (12.6-66.5)	32.1 (10.8-52.9)	47.6 (15.5-48.7)	0.556
BUN (mmol/L)	9.52 (5.21-18.23)	8.96 (5.00-16.3)	9.75 (4.75-18.92)	8.96 (4.95-14.21)	9.57 (5.56-15.54)	0.933
Creatinine (μmol/L)	318.31 (212.12-421.2)	256.42 (189.92-356.1)	265.26 (190.50-366.12)	168.01 (107.12-182.9)	247.58 (115.22-357.23)	0.433
AST (μkat/L)	0.33 (0.29-0.59)	0.48 (0.21-0.67)	0.37 (0.20-0.57)	0.27 (0.21-0.52)	0.35(0.25-0.58)	0.100
ALT (μkat/L)	0.38 (0.26-0.55)	0.45 (0.33-0.59)	0.40 (0.33-0.59)	0.25 (0.22-0.58)	0.42 (0.32-0.52)	0.408
Total cholesterol (mmol/L)	5.59 (4.13-6.83)	4.54 (4.01-5.87)	5.97 (4.34-6.11)	5.35 (4.25-6.32)	5.12 (4.32-5.99)	0.144
Triglyceride (mmol/L)	1.60 (1.07-2.73)	1.43 (0.91-2.10)†	2.29 (1.76-2.89)	2.28 (1.76-2.83)	1.90 (1.45-2.21)	0.025*
LDL (mmol/L)	3.93 (3.10-4.69)	3.18 (2.99-4.56)	4.24 (3.42-4.99)	3.36 (2.70-4.51)	3.98 (2.98-4.91	0.628
HDL (mmol/L)	1.06 (0.92-1.29)	0.91 (0.88-1.11)	1.03 (0.91-1.31)	1.22 (1.09-1.29)	1.09 (0.99-1.31)	0.628
Uric acid (μmol/L)	398.2 (298.2-411.2)	410.4 (299.1-460.3)	380.6 (265.2-398.3)	485.8 (373.1-499.2)	392.5 (279.3-443.1)	0.490

Sodium (mmol/L)	131.6 (130.2-138.5)	133.4 (129.3-139.2)	132.9 (129.5-136.9)	137.9 (132.9-141.6)	134.9 (132.7-140.6)	0.821
Potassium (mmol/L)	3.8 (3.5-4.3)	3.9 (3.5-4.5)	3.8 (3.4-4.2)	4.2 (3.4-4.9)	4.0 (3.5-4.6)	0.824
Calcium (mmol/L)	2.14 (2.09-2.72)	2.11 (1.99-2.67)	1.99 (1.96-2.76)	1.92 (1.91-2.11)	2.12(1.98-2.32)	0.900
Phosphate (mmol/L)	1.18 (1.13-1.55)	0.99 (0.96-0.1.66)	1.10 (0.95-1.94)	1.06 (0.89-2.10)	1.01 (0.91-2.02)	0.921
Albumin (g/L)	22 (19-29)	20 (17-28)	21 (19-29)	18 (15-24)	22 (18-31)	0.876
Total protein, g/L	52 (39-56)	41 (35-55)	38 (33-51)	32 (21-49)	49 (31-59)	0.425
ANA (%)	0 (0)	0 (0)	0 (0)	6 (54.5)[†]	0 (0)	<0.001†
VDRL (%)	0 (0)	0 (0)	1 (0.9)	2 (18.2)[†]	1 (2.6)	0.001†
Fibrinogen (g/L)	4.73 (3.56-5.87)	7.32 (6.74-8.31)	3.97 (2.99-4.12)	3.42 (3.31-4.22)	4.65 (3.73-5.21)	0.084
Homocysteine (μmol/L)	11.9 (8.9-14.2)	14.6 (12.3-16.9)	12.3 (112.2-130.1)	9.7 (8.9-11.2)	13.0 (12.7-14.3)	0.707
Protein S (%)	121.2 (106.6-131.9)	118.1 (106.4-124.2)	99.1 (87.9-111.2)	104.0 (99.1-115.8)	120.0 (116.1-134.1)	0.083
Protein C (%)	120.7 (113.2-127.1)	115.2 (106.1-124.4)	117.6 (108.2-125.6)	103.8 (106.1-121.2)	107.1 (103.2-122.4)	0.547
Left ventricular ejection fraction (%)	58.2 (49.6-68.5)	58.5 (48.4-59.5)	61.2 (50.4-72.1)	55.1 (49.5-62.1)	58.4 (50.3-66.2)	0.215
Complication at admission						
Other infection (%)	7 (16.3)	1 (4.3)	5 (4.3)	1 (9.1)	3 (7.7)	0.132
Acute coronary syndrome (%)	1 (2.3)	1 (4.3)	0 (0)	0 (0)	1 (2.6)	0.376
Venous thromboembolism (%)	1 (2.3)	1 (4.3)	0 (0)	0 (0)	0 (0)	0.213
Pulmonary edema (%)	4 (9.3)	3 (13)	4 (3.4)	0 (0)	2 (5.1)	0.261
Glasgow coma scale score						

Upon admission	15 (15-15)	15 (13-15)	15 (15-15)	15 (15-15)	15 (15-15)	<0.001*
Upon discharge	15 (15-15)	15 (15-15)	15 (15-15)	15 (15-15)	15 (15-15)	<0.001*
modified Rankin scale score						
Upon admission	4 (3-5)	3 (3-4)	3 (2-3)	2 (2-4)	3 (3-5)	<0.001*
Upon discharge	4 (3-5)	3 (3-5)	3 (2-3)	2 (2-4)	3 (3-4)	<0.001*

BMI indicates body mass index; SBP, systolic blood pressure; DBP, diastolic blood pressure; WBC, white blood cells; INR, international normalized ratio; APTT/NC, activated partial thromboplastin time/normal control; hs-CRP, high-sensitivity C-reactive protein; ESR, erythrocyte sedimentation rate; BUN, blood urea nitrogen; AST, aspartate transaminase; ALT, alanine aminotransferase; LDL, low-density lipoprotein; HDL, high-density lipoprotein; ANA, antinuclear antibody; VDRL, the venereal disease research laboratory test.

Data are presented as median (interquartile range) or absolute numbers (percentage).

*P < 0.05, Kruskal-Wallis test; †P < 0.05, Chi-square test.

Supplementary Table 4. Demographic features and clinical courses of nephrotic syndrome patients with intracranial hemorrhage (additional data).

	Any intracranial hemorrhage (n=57)				
Types of hemorrhage	Intracerebral hemorrhage (n=45)	Subarachnoid hemorrhage (n=4)	Subdural hemorrhage (n=7)	Arteriovenous malformation (n=1)	*P* value
Location of hemorrhage (patients)	Cerebral hemisphere: 10, basal ganglia: 20, brainstem: 8, cerebellum: 7				
Complication at admission					
Pneumonia (%)	9 (20)	1 (25)	1(14.3)	0 (0)	0.930
Gastrointestinal bleeding (%)	8 (17.8)	1 (25)	0 (0)	0 (0)	0.596
Urinary tract infection (%)	10 (22.2)	2 (50)	1 (14.3)	0 (0)	0.518
Other infection (%)	2 (4.4)	0 (0)	0 (0)	0 (0)	0.907
Acute coronary syndrome (%)	1 (2.2)	0 (0)	0 (0)	0 (0)	0.965
Venous thromboembolism (%)	1 (2.2)	0 (0)	0 (0)	0 (0)	0.965
Pulmonary edema (%)	1 (2.2)	0 (0)	0 (0)	0 (0)	0.965

Data are presented as absolute numbers (percentage).

**P* < 0.05, Chi-square test.

Supplementary Table 5. Cox regression analysis of patient survival during the 30-day period, intracranial hemorrhage vs. ischemic stroke (detailed items).

	Univariate Cox regression		Multivariate Cox regression	
	HR (95% CI)	*P* value	HR (95% CI)	*P* value
Age	1.01 (0.98-1.04)	0.610		
Male	0.67 (0.29-1.58)	0.364		
Hypertension	1.21 (0.36-4.10)	0.762		
Diabetes mellitus	0.96 (0.40-2.33)	0.936		
Hyperlipidemia	0.22 (0.08-0.60)	0.003*		
Coronary artery disease	3.12 (1.26-7.72)	0.014*	4.19 (1.67-10.46)	0.002†
Congestive heart failure	2.58 (1.00-6.65)	0.050*		
Atrial fibrillation	1.00 (0.23-4.28)	0.996		
Old stroke	1.05 (0.43-2.61)	0.912		
Smoking	0.57 (0.08-4.25)	0.583		
End-stage renal disease with dialysis	2.20 (0.85-5.67)	0.103		
Pneumonia	3.66 (1.54-8.68)	0.003*		
Intracranial hemorrhage	7.33 (3.04-17.70)	<0.001*	10.68 (3.31-34.47)	<0.001†
Total anterior circulation syndrome	4.82 (1.62-14.34)	0.005*	17.65 (4.37-71.23)	<0.001†

HR indicates hazard ratio; CI, confidence interval.

**P* < 0.1 for the univariate Cox regression, and †*P* < 0.05 for the multivariate Cox regression.

Supplementary Table 6. Cox regression analysis of patient survival during the 30-day period in nephrotic syndrome patients with ischemic stroke (detailed items).

Variables	Univariate Cox regression, HR (95% CI)	*P* value	Multivariate Cox regression, HR (95% CI)	*P* value
Age	0.98 (0.94-1.03)	0.524		
Male	1.85 (0.37-9.17)	0.451		
Hypertension	0.58 (0.12-2.88)	0.506		
Diabetes mellitus	0.88 (0.21-3.69)	0.864		
Hyperlipidemia	0.18 (0.04-0.91)	0.037*		
Coronary artery disease	3.50 (0.84-14.64)	0.086*	24.58 (1.48-408.90)	0.026†
Congestive heart failure	3.83 (0.92-16.02)	0.066*		
Atrial fibrillation	1.33 (0.16-10.82)	0.789		
Hyperuricemia	0.68 (0.08-5.55)	0.721		
Old stroke	0.28 (0.04-2.30)	0.238		
Smoking	1.37 (0.17-11.17)	0.766		
TACS	16.41 (4.10-65.72)	<0.001*		
Pneumonia	8.34 (1.99-34.92)	0.004*		
Gastrointestinal bleeding	3.42 (0.69-16.96)	0.132		
Hemoglobin	0.82 (0.61-1.09)	0.161		
Hs-CRP	1.04 (1.02-1.06)	<0.001*		
Platelet	1.00 (0.99-1.01)	0.792		
Albumin	1.26 (0.73-2.18)	0.413		

HR indicates hazard ratio; CI, confidence interval; TACS, total anterior circulation syndrome; hs-CRP, high-sensitivity C-reactive protein.

*$P < 0.1$ for the univariate Cox regression, and †$P < 0.05$ for the multivariate Cox regression.

Supplementary Table 7. Cox regression analysis of patient survival during the 30-day period in nephrotic syndrome patients with intracranial hemorrhage (detailed items).

Variables	Univariate Cox regression, HR (95% CI)	*P* value	Multivariate Cox regression, HR (95% CI)	*P* value
Age	1.04 (0.99-1.08)	0.120		
Male	0.37 (0.12-1.12)	0.078*		
Hypertension	2.88 (0.37-22.13)	0.310		
Diabetes mellitus	1.54 (0.50-4.71)	0.448		
Hyperlipidemia	0.65 (0.18-2.37)	0.516		
Coronary artery disease	3.93 (1.20-12.85)	0.023*	5.49 (1.54-19.56)	0.009†
Congestive heart failure	1.98 (0.54-7.20)	0.301		
Atrial fibrillation	1.13 (0.15-8.66)	0.909		
Hyperuricemia	1.00 (0.13-7.69)	1.00		
Old stroke	2.42 (0.81-7.21)	0.112		
Intracerebral hemorrhage	0.96 (0.27-3.50)	0.955		
Subarachnoid hemorrhage	4.23 (1.15-15.52)	0.030*	6.32 (1.57-25.53)	0.010†
Smoking	0.05 (0.00-455.3)	0.734		
Pneumonia	1.91 (0.59-6.19)	0.284		
Gastrointestinal bleeding	0.89 (0.20-4.00)	0.875		
Hemoglobin	0.85 (0.66-1.09)	0.195		
Hs-CRP	1.00 (0.98-1.02)	0.812		
Platelet	1.00 (1.00-1.01)	0.157		
Albumin	0.98 (0.67-1.44)	0.925		

HR indicates hazard ratio; CI, confidence interval; hs-CRP, high-sensitivity C-reactive protein.

*$P < 0.1$ for the univariate Cox regression, and †$P < 0.05$ for the multivariate Cox regression.
